# 

**Published:** 1874-12

**Authors:** 


### CONTENTS.

### COMMUNICATIONS:

Introductory Address before the Class in the Ohio Dental College . . . . . . . . 493
Anæsthetics—Three Deaths from Chloroform and Ether 500
Filling Frail Teeth with Gold . . . . . . . 502
Remarks on the Advance in Practice and Use of Mechanical Appliances . . . . . . . . 504
Dentistry in the Exposition . . . . . . 510

### PROCEEDINGS OF SOCIETIES:

Michigan Dental Association . . . . . . 513
Proceedings of Mad River Valley Dental Society . 518
Dental Society . . . . . . . . . . 518
Experiment in Liquefying Gases . . . . . 520
Improvements in Working Rubber . . . . . 521

### EDITORIAL:

Success , . . . . . . . . . . 523
Failures . . . . . . . . . . . 525
Dental Vulcanite Controversy . . . . . . 526
Ohio Dental College Laboratory . . . . . 528
For Sensitive Dentine . . . . . . . . 530
Spongoid . . . . . . . . . . . 531
Notice . . . . . . . . . . . . 447
The Physician's Visiting List . . . . . . . 532

